# The genus *Ectonura* Cassagnau, 1980in South Africa (Collembola, Neanuridae, Neanurinae), with a key to South African Neanurinae

**DOI:** 10.3897/zookeys.136.1744

**Published:** 2011-10-13

**Authors:** Charlene Janion, Anne Bedos, Louis Deharveng

**Affiliations:** 1 Centre for Invasion Biology, Department of Botany and Zoology, Stellenbosch University, South Africa; 2 Museum National d'Histoire Naturelle, UMR7205 “Origine, Structure et Evolution de la Biodiversité”, Paris, France

**Keywords:** Taxonomy, new species, Western Cape, Fynbos

## Abstract

Two new species of Neanurinae (Collembola) are described from the Western Cape, South Africa: *Ectonura monochaeta* **sp. n.** and *Ectonura barrai* **sp. n.** *Ectonura monochaeta* **sp. n.** differs from other species in the genus by its strongly reduced chaetotaxy, and the lateral shift of dorso-internal chaetae on Abd. V and their integration in the tubercles (De+DL). *Ectonura barrai* **sp. n.** is similar to *Ectonura natalensis* (Womersley, 1934), but differs in chaetotaxic details and chaetal group arrangement. A key to the seven species of Neanurinae recorded from South Africa is given.

## Introduction

**Figure 1.**
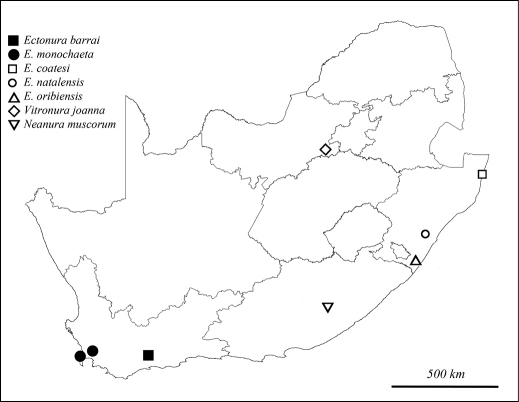
Distribution of Neanurinae recorded from South Africa.

Neanurinae Collembola are represented in tropical Africa by a large number of species in the tribe Paleonurini, and a single representative of the tribe Neanurini, the parthenogenetic species *Neanura muscorum* (Templeton, 1835). However, only a few areas have been sampled outside the mountain ranges of Eastern Africa (Cassagnau 1991, 1996, 2000; Weiner and Najt 1998). In South Africa, only three genera and five species of Neanurinae have been recorded so far (Fig. 1): *Neanura* MacGillivray, 1893 with *Neanura muscorum*; *Vitronura* Yosii, 1969 with *Vitronura joanna* (Coates, 1968), and *Ectonura* Cassagnau, 1980 with *Ectonura natalensis* (Womersley, 1934), *Ectonura oribiensis* (Coates, 1968) and *Ectonura coatesi* Barra, 1994. *Neanura muscorum* has been probably introduced from Europe, where all other species of the genus *Neanura* occur. The genus *Vitronura*, diversified in China, Sunda and western Pacific islands, has a single widespread species, *Vitronura giselae* (Gisin, 1950), that occurs both in the tropics and in gardens in Europe. Therefore, the presence of another distinct species isolated in South Africa requires confirmation, as *Vitronura joanna* is morphologically very close to *Vitronura giselae*. The genus *Ectonura*, limited to South Africa in the African continent, includes the only Neanurinae unambiguously native of South Africa; the genus is otherwise present in New Caledonia with 11 species (Deharveng and Bedos 2002), and an undescribed species is recorded from South Australia by Greenslade and Deharveng (1991).

Among the large amount of samples recently collected in the Western Cape Province in the frame of the Franco-South African PROTEA project “Uncovering Springtail Diversity in the South African Cape Floristic Region: a combined taxonomic and barcoding approach”, we retrieved representatives of the three cited genera, including new *Ectonura* species, as well as a single species of a fourth genus, *Paleonura* Cassagnau, 1982. This confirms that South Africa fauna of Neanurinae is particularly poor, compared to that of East African mountains or Madagascar (Cassagnau 1996, 2000), or other gondwanian territories such as Australia (Greenslade and Deharveng 1991). Its richness in Neanurinae is actually similar to that of southern America subtemperate areas, where *Neanura muscorum* also occurs, together with a few endemic *Paleonura* (Cassagnau and Oliveira 1990).

*Ectonura* is therefore the most diversified genus of Neanurinae in southern Africa. Several undescribed species were present in our samples, mostly as isolated specimens. Two of them were collected in sufficient number and are described in this paper: *Ectonura monochaeta* sp. n. from Table Mountain and *Ectonura* *barrai* sp. n. from Grootvadersbosch, both located in the Western Cape Province.

### Abbreviations used

Type deposit – IMIziko Museum (Cape Town, South Africa); MNHNMuseum National d'Histoire Naturelle (Paris, France).

Chaetal arrangement and notation follow Deharveng (1983) and Smolis (2008).

Abbreviations used in species descriptions and tables:

Tubercles and chaetal groups – Afantenno-frontal; CLclypeal; Dedorso-external; Didorso-internal; DLdorso-lateral; Llateral; Ococular; Sosubocular; VLventro-lateral; Veventro-external; Viventro-internal; Agantegenital; Ananal.

Appendages – Cxcoxa; Fefemur; Fufurcal rest; Scx2subcoxa 2; Titatibiotarsus; Trtrochanter; VTventral tube.

Types of chaetae – Mmacrochaeta; memesochaeta; mimicrochaeta; SS-chaeta (“sensillum” *auct*.); mss-microchaeta.

Others – ommaommatidia; Abd.abdominal segment; Ant.antennal segment; Th.thoracic segment.

## Key to South African species of Neanurinae

**Table:** 

1	Three eyes on each side of the head, body bluish in colour	*Neanura muscorum* (Templeton, 1835) (temperate cosmopolite)
–	Two eyes on each side of the head, body not blue	2
2	Central area of head with 3 tubercles	*Vitronura joanna* (Coates, 1968) (uncertain status)
–	Central area of head with 2 tubercles	*Ectonura* 3
3	Dorso-internal tubercle of tergites from Th. II to Abd. IV with at least two chaetae	4
–	Dorso-internal tubercle of tergites with one chaeta from Th. II to Abd. IV	*Ectonura monochaeta* sp. n.
4	Chaeta A absent on head	5
–	Chaeta A present on head	6
5*	Modified chaetae Ag on Abd. V of the male much shorter than lateral Ag, very thick, truncated, apically ciliated	*Ectonura oribiensis* (Coates, 1968) (endemic)
–	Modified chaetae Ag on Abd. V of the male as long as lateral Ag, thin, pointed, with several unequal distal cilia	*Ectonura coatesi* Barra, 1994 (endemic)
6	Five chaetae on each central group of chaetae on head (1/2Af+Oc), chaeta D absent; one chaeta Di on Abd. V (Di2 absent); tubercles Di of Abd. V fused on axis	*Ectonura natalensis*(Womersley, 1934) (endemic)
–	Six chaetae on each central group of chaetae on head (1/2Af+Oc), chaeta D present; two chaetae Di on Abd. V (Di2 present); tubercles Di of Abd. V separate	*Ectonura barrai* sp. n.

* The only differential character separating unambiguously *Ectonura coatesi* from *Ectonura oribiensis* is the morphology of modified chaetae Ag in the male.
            

## Taxonomy

***Ectonura* Cassagnau, 1980**

**Type species:** *Achorutes natalensis* Womersley, 1934 (Natal, South Africa)

### Ectonura monochaeta sp. n.

urn:lsid:zoobank.org:act:FBCFC562-E830-4085-B711-3BCF1F4758DD

http://species-id.net/wiki/Ectonura_monochaeta

Figs 2 3 Table 1 

#### Type material.

Holotype female on slide. South Africa: Western Cape, Cape Town, Table Mountain National Park, 10/03/2009, native broadleaved forest, sieving of litter and extraction on Berlese funnel, Louis Deharveng and Anne Bedos leg (SAF-141).

8 paratypes on slides (3 males, 4 females, 1 juvenile) and more than 50 in alcohol, same data as holotype — 1 male on slide, ibid, Table Mountain, collapse of New Year cave system, 07/03/2009, native forest, litter, Berlese extraction, Louis Deharveng and Anne Bedos leg (SAF-129) — 1 male and 1 female on slides, ibid, Table Mountain, in a collapse, 10/03/2009, native forest, soil, Berlese extraction, Louis Deharveng and Anne Bedos leg (SAF-139) — 1 male and 1 female on slides, 5 specimens in alcohol, ibid, Table Mountain, Inchuk cave entrance, 10/03/2009, native forest, litter, Berlese extraction, Louis Deharveng and Anne Bedos leg (SAF-144).

#### Type deposition.

Holotype, 6 paratypes on slides (3 males, 3 females) and 25 paratypes in alcohol in IM; 7 paratypes on slides (3 males, 3 females, 1 juv.) and 25 paratypes in alcohol in MNHN.

#### Non-type material.

1 male on slide, 7 specimens in alcohol. South Africa: Western Cape, Stellenbosch, Jonkershoek Nature Reserve, Sosys trail, 12/03/2008, forest litter, Berlese extraction, Louis Deharveng and Anne Bedos leg (SAF-071) — 1 juvenile and 2 females on slides, ibid, Jonkershoek Nature Reserve, Sosys trail, 12/08/2010, litter, Charlene Janion leg (RSA10_JNK026 and RSA10_JNK032, 33°59.758'S, 18°57.156'E) — 1 male, 1 female and 1 juvenile on slides, about 130 in alcohol, ibid, Fish Hoek, Kalk Bay, Echo Valley forest, 05/11/2010, decaying wood of yellowwood, Berlese extraction, Louis Deharveng and Anne Bedos leg (SAF-196).

#### Description.

**Figure 2.**
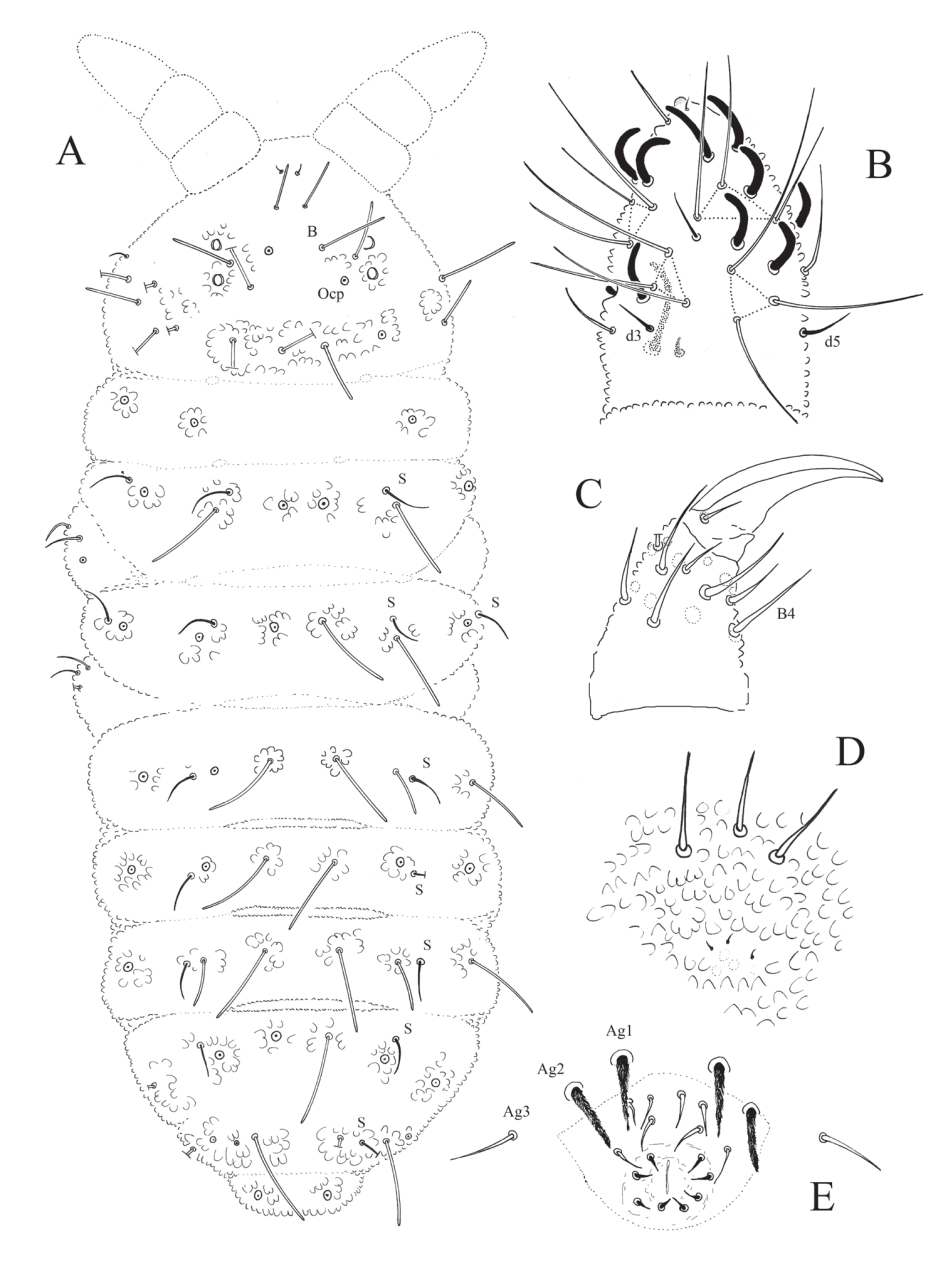
*Ectonura monochaeta* sp. n.; **A** dorsal chaetotaxy **B** Ant. III-IV dorsal side **C** tibiotarsus and praetarsus of leg I **D** furcal rest **E** male genital area.

**Figure 3.**
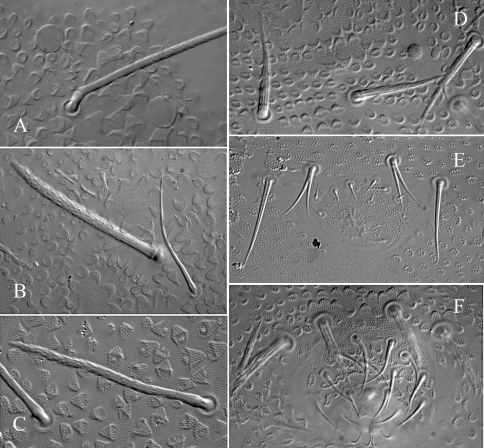
*Ectonura monochaeta* sp. n.; **A** Ocular plate with macrochaeta Ocm **B** macrochaeta De1 on Th. II **C** chaeta B on head **D** chaetae Ve posterior of Abd. IV in a paratype male adult **E** genital plate of a male juvenile from Jonkershoek **F** genital plate of a male adult.

Length 0.82 – 1.1 mm (males) and 0.75 – 0.85 mm (females). Colour white in alcohol, yellow alive (SAF-196 sample). Eyes 2+2, unpigmented, small (diameter about 1.5–1.8 times that of Ocm socket, Fig. 3A). Habitus similar to *Paleonura* (Fig. 2A). No cryptopygy. Secondary granules rather large (the size of a mesochaeta socket). Dorsal tubercles visible but poorly delimited except on Abd. V-VI, indicated by secondary granules enlarged and irregularly arranged, without clearly developed tertiary granules. No reticulations. No plurichaetosis. Most ordinary dorsal chaetae are macrochaetae of similar length and morphology, basally swollen, straight, cylindrical, long, thick, covered in their 2/3 distal of numerous minute scales, distally sheathed, rounded apically (Figs 2A, 3A, B, C). Some dorsal mesochaetae shorter, bent, acuminate, smooth or weakly rugose on the lateral area of head, on tubercles L of tergites and on Abd. VI. No dorsal microchaetae. S-chaetae thin, 2/3 as long as or slightly shorter than closest macrochaeta (Figs 2A, 3B).

Ant. I with 7 chaetae, Ant. II with 11 chaetae, Ant. III with 16 or 17 chaetae (chaetae d4 and d5 or only d4 absent). Ant. IV organite as a short thick rod; apical bulb simple, low and fused to Ant. IV tip (Fig. 2B). Buccal cone moderately elongated. Maxilla styliform, mandible thin and bidentate with distal tooth subdivided in 2 or 3 minute cilia. Labrum elongate, rounded apically, with ventral sclerifications arc-like. Labral formula 0/2,4. Labium with 4 basal, 3 distal and 3 lateral chaetae, and 2 minute sphaerical x papillae (as in *Ectonura barrai* sp. n., Fig. 5B).

Head chaetotaxy as in Table 1A and Fig. 2A. Head with 6 chaetal groups: CL, 2 (½ Af + Oc), (2 Di, 2 De), 2 (DL, L, So). Central area with B, F, G, Ocm and Ocp. Macrochaeta Ocm internal to ocular line, equally distant to omma or slightly closer to anterior omma; Ocp macrochaeta internal to and at level of posterior omma; Oca absent (Fig. 3A). Posterior area with a very faint tubercle and only 2+2 macrochaetae (Di1 and De1). Five chaetae Vi ventrally on head (Vi5 absent).

**Figure 4.**
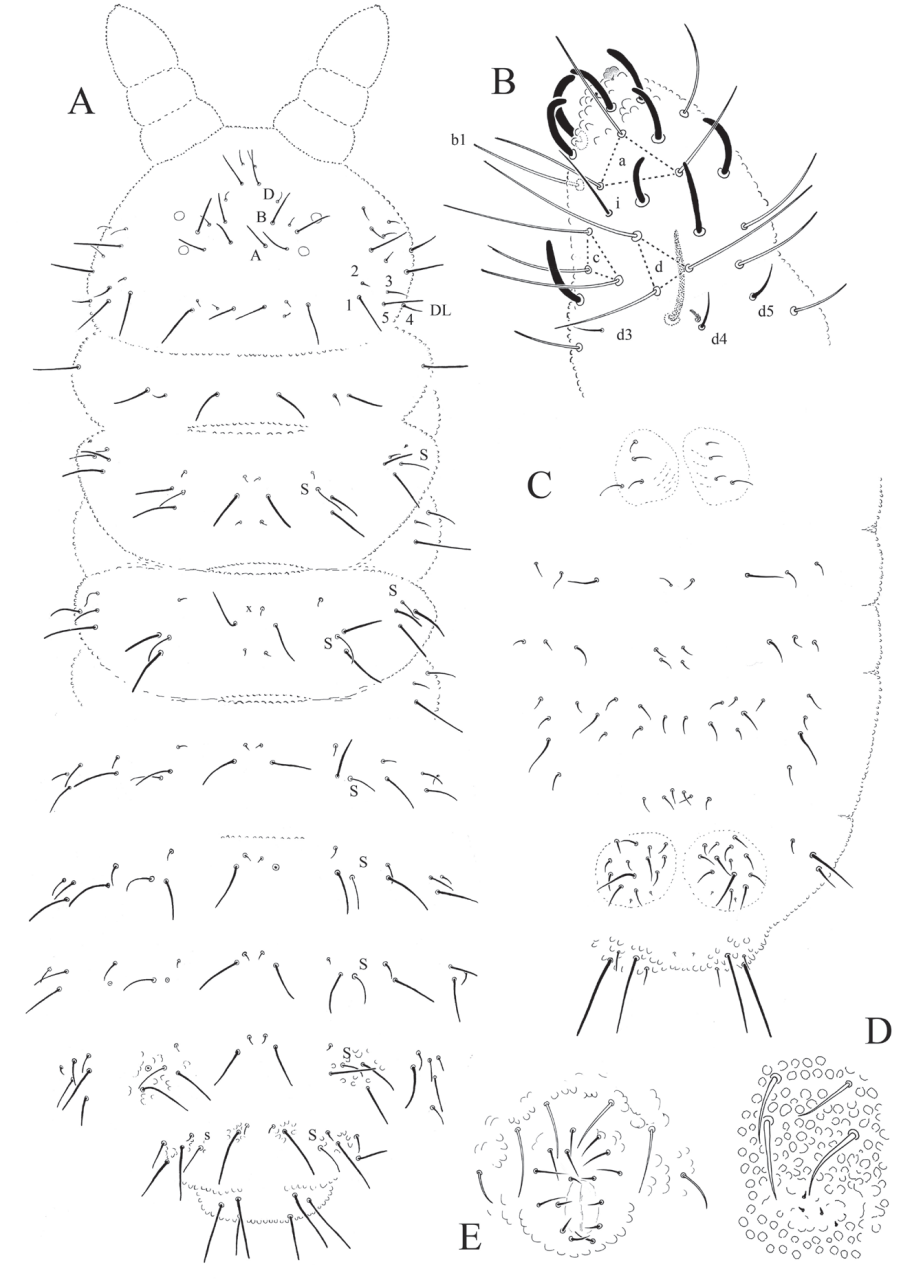
*Ectonura barrai* sp. n.; **A** dorsal chaetotaxy (tubercles not represented except on Abd. IV-V; x, chaeta Di2 absent unilaterally on Th. III) **B** Ant. III-IV dorsal side **C** ventral chaetotaxy of abdomen **D** Furcal rest **E** male genital plate.

Tergite chaetotaxy as in Table 1B and Fig. 2A. Chaeta Di absent on Th. I. Tubercles De and DL separate on Abd. IV. Tubercle L of Abd. IV shift ahead the tubercle line Di-De-DL. Tubercles Di, De and DL fused on Abd. V on each side of axis. Tubercle Di of Abd. V with Di1 macrochaeta, Di2 and Di3 absent. Abd. VI not or hardly bilobed, with strong secondary granules, present even on the axis. S-chaetotaxic formula: 2+ms, 2/11111. Ventral chaetotaxy similar to that of *Ectonura barrai* sp. n., except the furcal rest in some specimens (Fig. 4C). Secondary sexual characters well developed in the adult male consists of chaetae Ag1 and Ag2 of Abd. V strongly thickened and serrated (Figs 2E, 3F), and chaetae of furcal rest, some Ve of Abd. IV (Fig. 3D), sometimes Ag3 of Abd. V (Fig. 3F), and 3+3 Ve of Abd. VI serrated but less strongly. In a male juvenile from Jonkershoek, chaetae Ag1 were bifid (Fig. 3E).

Microchaetae of furcal rest smaller than secondary granules, often unconspicuous (Fig. 2D). Leg chaetotaxy given in Table 1C. Tita without chaeta M and with chaetae B4-B5 short, not longer than other long chaetae of Tita (Fig. 2C). Claw untoothed, not striated in its basal part, and devoid of secondary granulation.

#### Derivatio nominis.

The species name refers to its reduced chaetotaxy of dorso-internal tubercles of tergites, which bear only one chaeta from Th. II to Abd. IV (2 or 3 in other species of the genus).

#### Ecology.

All known localities of *Ectonura monochaeta* sp. n. belong to the Southern Afrotemperate Forest vegetation type. The species is common in this habitat, typically found in the Western Cape, but absent in shrub formations of the fynbos. The distribution ranges from Table Mountain National Park to Jonkershoek Nature Reserve, Stellenbosch. The species is mixed in Stellenbosch with another undescribed species of *Ectonura*.

#### Discussion.

The new species *Ectonura monochaeta* is unique in the genus by the lateral shift of dorso-internal chaetae on Abd. V and their integration in the tubercles (De+DL). Such a lateral shift is only know in *Ectonura paralata* Deharveng, Weiner & Najt, 1997 from New Caledonia, but less marked and without integration of Di chaetae in (De+DL). By other chaetotaxic characters (Di2 and De2 present on head and on tergites of Th. II-Abd. IV; D, E, OcA present on head) and tubercle arrangement (tubercle Di not developed, others as large flat plates), *Ectonura paralata* is however only remotely related to our species. *Ectonura monochaeta* is also distinct from other species of the genus *Ectonura* by the strong reduction of its chaetotaxy: absence of several chaetae on head (A, O, C, D, E, Oca), absence of Di2, De2 and DL2 on tergites, only 2+2 dorsal chaetae on Th. I, and only 6+6 chaetae on Abd. VI.

The lateral shift of Di on Abd. V is one of the characteristic feature of two genera, the monotypic genus *Zelandanura* Deharveng & Wise, 1991 from Campbell Island and *Pronura* Delamare Debouteville, 1953 which is highly diversified in Africa and in Asia. *Zelandanura* differs from *Ectonura* by the fusion in one plate of all tubercles of the central area of head, and by the fusion of Di tubercles on the axis on Abd. IV. Contrary to *Ectonura*, chaetae of the central area of head are not separated in two groups on both side of the axis in *Pronura*.

**Table 1.** Chaetotaxy of *Ectonura monochaeta* sp. n.

**A-Cephalic chaetotaxy**					
**Group of chaetae**	**Tubercle**	**Number of chaetae**	**Type of chaetae**	**Chaetae**	
CL	(-)	2	M	F	
		2	me	G	
1/2Af+Oc	(+)	3	M	B, Ocm, Ocp	
2Di, 2De	(+)	2+2	M	Di1, De1	
DL, L, So	(-)	5	M	DL1, DL5, L1, L4, So1	
		4	me	So3 to 6	
**B-Tergite chaetotaxy**					
	**Di**	**De**	**DL**	**L**	
Th. I	-	1	1	-	
Th. II	1	1+S	1+S+ms	3	
Th. III	1	1+S	1+S	3	
Abd. I	1	1+S	1	2	
Abd. II	1	1+S	1	2	
Abd. III	1	1+S	1	2	
Abd. IV	1	1+S	1	5	
Abd. V	3+S			2	
Abd. VI	6*				
**C-Leg chaetotaxy**					
	**Scx2**	**Cx**	**Tr**	**Fe**	**Tita**
Leg I	0	3	6	13	18
Leg II	2	7	6	12	18
Leg III	2	8	6	11	17
**D-Sternite chaetotaxy**					
Abd. I	VT: 4				
Abd. II	Ve: 4	(Ve1 present)			
Abd. III	Ve: 3	Fu: 3-5me+3mi			
Abd. IV	Ve: 7	VL: 4			
Abd. V	Ag: 3	VL: 1, with L			
Abd. VI	Ve: 12-13	An: 2 mi			

* the ventralmost pair of chaetae is replaced by a unique uneven chaeta in some specimens

### Ectonura barrai sp. n.

urn:lsid:zoobank.org:act:E0C421EE-4014-41F9-9A71-F23C8A7A6AEB

http://species-id.net/wiki/Ectonura_barrai

Figs 4 [Fig F5] 6 Table 2 

#### Type material.

Holotype male on slide. South Africa: Western Cape, Grootvadersbosch Nature Reserve, Heidelberg, 24/08/2010, Southern Afrotemperate Forest vegetation, in litter, extraction on Berlese funnel, Charlene Janion leg (RSA10_GVB009, 33°59.167'S, 20°48.639'E).

1 male paratype on slide, same data as holotype — 2 paratypes on slides (1 male, 2 juveniles) and 5 paratypes in alcohol, ibid, Grootvadersbosch Nature Reserve, Heidelberg, 24/08/2010, same habitat, extraction on Berlese funnel, Charlene Janion leg (RSA10_GVB008, 33°58.964'S, 20°48.524'E)

#### Type deposition.

Holotype and 4 paratypes (1 male and 1 juvenile on slides, 2 in alcohol) in IM; 5 paratypes (1 male and 1 juvenile on slides, 3 in alcohol) in MNHN.

#### Description.

**Figure 5. F5:**
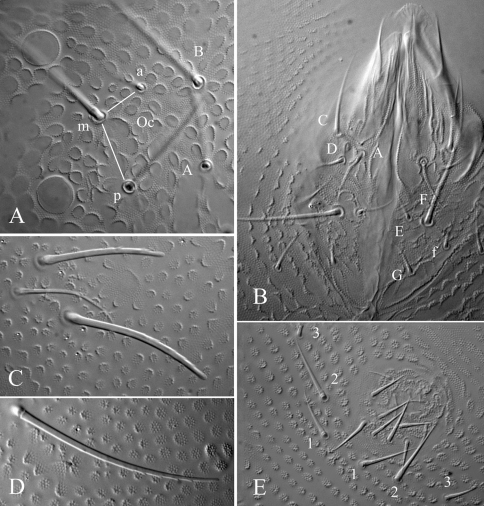
*Ectonura barrai* sp. n.; **A** ocular plate **B** Labium **C** chaetal group De on Th. III (without the De3 microchaeta) **D** macrochaeta DL on Abd. I **E** male genital plate.

**Figure 6.**
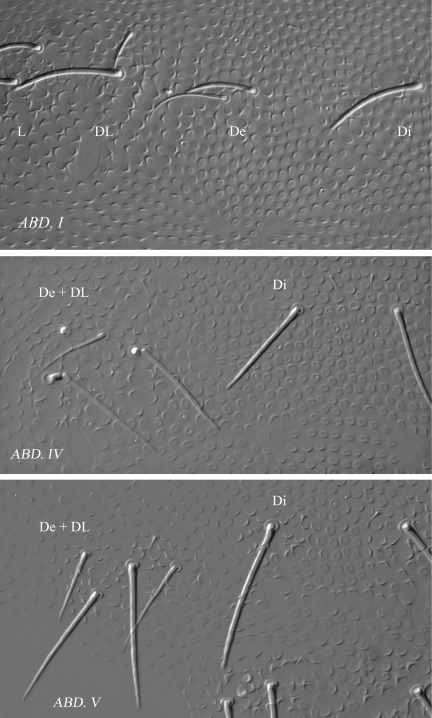
*Ectonura barrai* sp. n.; chaetal groups and faint tubercles Di, De and DL on Abd I, Abd. IV and Abd. V.

Length 1.1–1.3 mm (males). Colour white in alcohol. Eyes 2+2, unpigmented, rather large (diameter about 3 times that of Ocm socket, Fig. 5A). Habitus similar to *Paleonura* (Fig. 4A). No cryptopygy. Secondary granules rather large (the size of a mesochaeta socket). Dorsal tubercles not clearly delimited, only indicated by secondary granules irregularly arranged, without tertiary granules. No reticulations. No plurichaetosis. Ordinary dorsal chaetae differentiated in macrochaetae, mesochaetae and microchaetae (Figs 4A, 6). Dorsal macrochaetae basally swollen, straight or slightly bent, subcylindrical, long, moderately thick, with minute scales sparsed apparently unilaterally, in their distal half, distally sheathed, rounded apically (Figs 5C, D, 6). Dorsal mesochaetae shorter, acuminate to blunt, smooth or weakly rugose. Dorsal microchaetae thin, smooth, less than 1/5 of macrochaetae, present on all tergites (Di2 from Th. II to Abd. V, De3 on Th. II-III, and De2 on Abd. I-V). S-chaetae thin, smooth, acuminate, 2/3 as long as or slightly shorter than closest macrochaeta (Figs 5C, 6).

Ant. I with 7 chaetae, Ant. II with 11 chaetae, Ant. III with 18 chaetae (chaetae d4 and d5 present). Ant. IV organite as a very short thick rod; apical bulb feebly trilobed, fused to Ant. IV tip (Fig. 4B). Buccal cone moderately elongated. Maxilla styliform, mandible thin and bidentate with distal tooth subdivided in 2 or 3 minute cilia. Labrum elongate, rounded and finely denticulated apically, with ventral sclerifications arc-like distally. Labral formula 0/2,4. Labium with 4 basal, 3 distal and 3 lateral chaetae, and 2 minute sphaerical x papillae (Fig. 5B).

Head chaetotaxy as in Table 2A and Fig. 4A. Head with 9 chaetal groups: CL, 2 (½ Af + Oc), 2 DL, 2 (L, So), 2 (Di, De) on very faint tubercles hardly separated on axis. Central area with A, B, D, F, G, Oca, Ocm and Ocp (alternatively, Oca might be homologous of chaeta E). Macrochaeta Ocm internal to ocular line, equally distant from omma; Ocp macrochaeta internal to and at level of posterior omma; Oca antero-internal to Ocm (Fig. 5A). Five chaetae Vi ventrally on head (Vi5 absent).

Tergite chaetotaxy as in Table 2B and Figs 4A and 6. Chaeta Di present on Th. I. Tubercles De and DL fused on Abd. IV. Tubercle L of Abd. IV slightly shift ahead the tubercle line Di-De-DL. Tubercles De and DL fused on Abd. V, separated from Di. Tubercle Di of Abd. V with Di1 macrochaeta, Di2 microchaeta and Di3 absent. Abd. VI not or hardly bilobed, with 1+1 areas of slightly enlarged and irregularly arranged secondary granules. S-chaetotaxic formula: 2+ms, 2/11111. No modified chaetae in male (Figs 4E, 5E).

Microchaetae of furcal rest minute and thick, smaller than secondary granules (Fig. 4D). Leg chaetotaxy given in Table 2C, similar to that of *Ectonura monochaeta* sp. n. (Fig. 2C). Tita without chaeta M and with chaetae B4-B5 short, not longer than other long chaetae of Tita. Claw untoothed, not striated in its basal part, and devoid of secondary granulation.

#### Derivatio nominis.

This species is named in honour of Jean-Auguste Barra, for his important contribution to the knowledge of South African Collembola.

#### Ecology.

This species was collected in the yellowwood forest leaf litter of Grootvadersbosch Nature Reserve. This is a remnant forest of the larger Tsitsikamma Forest Reserve situated 300 km to the south. The forest consists of indigenous trees such as yellowwood, ironwood and stinkwood.

#### Discussion.

*Ectonura barrai* sp. n. is similar to *Ectonura natalensis* in its relatively complete chaetotaxy, but different in several details of chaetal arrangement. Based on the redescription of Coates (1968), *Ectonura natalensis* has a more reduced chaetotaxy than *Ectonura barrai* sp. n.: absence of meso/microchaetae D, Di2 and De2 on head, absence of De2 on Th. I, absence of De3 and DL3 on Th. II-III, absence of Di2 on Abd IV-V. Conversely, chaetae Di2, De2 and DL2 on Abd. I-III of *Ectonura natalensis* are much larger than homologous chaetae of *Ectonura barrai* sp. n. (macrochaetae or large mesochaetae versus short mesochaetae). Chaetal groups De and DL of Abd. IV are separate in *Ectonura natalensis* versus fused in *Ectonura barrai* sp. n., and chaetal groups Di of Abd. V are fused on axis in *Ectonura natalensis* versus separate in *Ectonura barrai* sp. n. The unusual arrangement of S-chaetae on Ant. IV figured by Coates (1968) is probably an erroneous interpretation, and not considered here as a valid differential character.

**Table 2.** Chaetotaxy of *Ectonura barrai* sp. n.

A-Cephalic chaetotaxy					
Group of chaetae	Tubercle	Number of chaetae	Type of chaetae	Chaetae	
CL	(-)	2	M	F	
		2	me	G	
1/2Af+Oc	(+)	4	M	A, B, Ocm, Ocp	
		2	me	D, Oca	
Di, De	(+)	2	M	Di1, De1	
		2	mi	Di2, De2	
DL	(+)	2	M	DL1, DL5	
		2	me	DL3, DL4	
		1	mi	DL2	
L, So	(-)	3	M	L1, L4, So1	
		5	me	L2, So3 to 6	
B-Tergite chaetotaxy					
	Di	De	DL	L	
Th. I	1	2	1	-	
Th. II	2-3	3+S	3+S+ms	3	
Th. III	2-3	3+S	3+S	3	
Abd. I	2	2+S	2	3	
Abd. II	2	2+S	2	3	
Abd. III	2	2+S	2	3-4	
Abd. IV	2	4+S		6	
Abd. V	2	4+S		3	
Abd. VI	7				
C-Leg chaetotaxy					
	Scx2	Cx	Tr	Fe	Tita
Leg I	0	3	6	13	18
Leg II	2	7	6	12	18
Leg III	2	8	6	11	17
D-Sternite chaetotaxy					
Abd. I	VT: 4				
Abd. II	Ve: 4	(Ve1 present)			
Abd. III	Ve: 3	Fu: 4me+4mi			
Abd. IV	Ve: 7	VL: 4			
Abd. V	Ag: 3	VL: 1, with L			
Abd. VI	Ve: 12-13	An: 2 mi*			

* those of upper valve shift close to the chaetae of Abd. VI

## Supplementary Material

XML Treatment for Ectonura monochaeta

XML Treatment for Ectonura barrai

## References

[B1] Barra JA (1994) Nouveaux Collemboles Poduromorphes de la Province du Natal (Rép. Sud Africaine) (Insecta : Collembola)*.* Journal of African Zoology 108: 181-189

[B2] Cassagnau P (1991) *Camerounura* n.g., un Collembole Neanurinae endémique du Mount Cameroun. Revue d'Ecologie et de Biologie du Sol 28 (2): 221-224

[B3] Cassagnau P (1996) Collemboles Paleonurini primitifs d'Afrique et de Madagascar. Annales de la Société entomologique de France 32 (2): 121-161

[B4] Cassagnau P (2000) Sur quelques Paleonurini d'Afrique Orientale (Collembola : Neanurinae). Annales de la Société entomologique de France 36 (2): 143-155

[B5] Cassagnau P Oliveira EP (1990) Les Collemboles Neanurinae d'Amérique du Sud. Bulletin de la Société d'Histoire naturelle de Toulouse 126: 19-23

[B6] Coates TJ (1968) The Collembola from South Africa – I : The Genus *Neanura*. The Journal of the Entomological Society of southern Africa 31: 185-195

[B7] Deharveng L (1983) Morphologie évolutive des Collemboles Neanurinae, en particulier de la lignée néanurienne. Travaux du Laboratoire d'Écobiologie des Arthropodes édaphiques, Toulouse 4: 1-63

[B8] Deharveng L Bedos A (2002) Nouveaux *Ectonura* de Nouvelle-Calédonie (Collemboles: Neanuridae). In: Zoologia Neocaledonica 5 Systématique et endémisme en Nouvelle-Calédonie.Mémoires du Muséum national d'Histoire naturelle de Paris 187: 91-102

[B9] Greenslade P Deharveng L (1991) *Phradmon*, a new genus of Paleonurini (Collembola: Neanuridae) from Australia, with a key to the genera from southern regions and notes on *Pronura*. Invertebrate Systematics 5: 837-854 doi: 10.1071/IT9910837

[B10] Smolis A (2008) Redescription of four Polish *Endonura* Cassagnau, 1979 (Collembola, Neanuridae, Neanurinae), with a nomenclature of the ventral chaetae of antennae. Zootaxa 1858: 9-36

[B11] Weiner WM Najt J (1998) Collembola (Entognatha) from East Africa. European Journal of Entomology 95: 217-237

[B12] Womersley H (1934) On some Collembola-Arthropleona from South Africa and Southern Rhodesia. Annals of the South African Museum 30: 441-475

